# An NIRS-based assay of chemical composition and biomass digestibility for rapid selection of Jerusalem artichoke clones

**DOI:** 10.1186/s13068-018-1335-1

**Published:** 2018-12-19

**Authors:** Meng Li, Siyang He, Jun Wang, Zuxin Liu, Guang Hui Xie

**Affiliations:** 1 College of Agronomy and Biotechnology, China Agricultural University, Beijing, 100193 China; 2 National Energy R&D Center for Non-food Biomass, China Agricultural University, Beijing, 100193 China; 3 Chinese Academy of Agricultural Engineering Planning and Design, Beijing, 100125 China

**Keywords:** Jerusalem artichoke, Chemical composition, Chemical pretreatment, Biomass digestibility, Near-infrared spectroscopy, Grey relational grade analysis

## Abstract

**Background:**

High-throughput evaluation of lignocellulosic biomass feedstock quality is the key to the successful commercialization of bioethanol production. Currently, wet chemical methods for the determination of chemical composition and biomass digestibility are expensive and time-consuming, thus hindering comprehensive feedstock quality assessments based on these biomass specifications. To find the ideal bioethanol feedstock, we perform a near-infrared spectroscopic (NIRS) assay to rapidly and comprehensively analyze the chemical composition and biomass digestibility of 59 Jerusalem artichoke (*Helianthus tuberosus* L., abbreviated JA) clones collected from 24 provinces in six regions of China.

**Results:**

The distinct geographical distribution of JA accessions generated varied chemical composition as well as related biomass digestibility (after soluble sugars extraction and mild alkali pretreatment). Notably, the soluble sugars, cellulose, hemicellulose, lignin, ash, and released hexoses, pentoses, and total carbohydrates were rapidly and perfectly predicted by partial least squares regression coupled with model population analyses (MPA), which exhibited significantly higher predictive performance than controls. Subsequently, grey relational grade analysis was employed to correlate chemical composition and biomass digestibility with feedstock quality score (FQS), resulting in the assignment of tested JA clones to five feedstock quality grades (FQGs). Ultimately, the FQGs of JA clones were successfully classified using partial least squares-discriminant analysis model coupled with MPA, attaining a significantly higher correct rate of 97.8% in the calibration subset and 91.1% in the validation subset.

**Conclusions:**

Based on the diversity of JA clones, the present study has not only rapidly and precisely examined the biomass composition and digestibility with MPA-optimized NIRS models but has also selected the ideal JA clones according to FQS. This method provides a new insight into the selection of ideal bioethanol feedstock for high-efficiency bioethanol production.

**Electronic supplementary material:**

The online version of this article (10.1186/s13068-018-1335-1) contains supplementary material, which is available to authorized users.

## Background

In recent years, fossil fuels consumption and greenhouse gas emissions have increased dramatically in step with rapid global industrialization, especially in China. Compared with developed economies, energy consumption in China has increased considerably in 2016 [1], accounting for 23% of global energy consumption and 27% of increased global energy demand [2]. In addition, estimated carbon emissions in China have grown by more than 75% since 2004 [2]. Consequently, the combination of China’s huge base energy consumption rate and its dramatically increasing energy demand is forcing its government to address energy needs and find sources of cleaner energy [1]. Bioenergy derived from lignocellulosic biomass currently holds great promise for addressing these energy and environmental concerns, due to large biomass reserves, resource reproducibility, low resource replacement costs, and low bioenergy production impact on carbon balance [3]. Moreover, lignocellulosic biomass is transformable into various forms of energy, such as briquettes, biogas, and bioethanol, making it an ideal substitute for fossil fuels [4–6].

Jerusalem artichoke (*Helianthus tuberosus* L., abbreviated JA), a perennial crop related to the sunflower, is widely distributed and cultivated in China and exhibits cold resistance, drought resistance, and salt tolerance [6–9]. Recently, numerous studies have demonstrated JA to be one of the most promising bioenergy crops for bioethanol production [6]. Indeed, JA tuber contains a considerable amount of inulin that is easily fermented into biofuel [10], while JA stem contains an abundance of lignocellulose that is available post-harvest [9, 11]. Paradoxically, despite a long history of JA tuber use for biofuel production, JA stem has been largely overlooked for this application and investigation of its use as feedstock for bioethanol production is warranted [6, 11, 12]. Principal chemical components of JA stem include soluble sugars, cellulose, hemicellulose, lignin, and ash, which vary widely among cultivated varieties [13]. Notably, variability in biomass major chemical constituents and physical structural attributes can lead to biomass recalcitrance when these attributes act in concert to negatively influence lignocellulosic biomass usability [14, 15]. Therefore, both principal chemical composition and biomass digestibility as feedstock quality specifications should be considered together for selecting ideal JA accessions. In addition, the ideal bioethanol feedstock could be identified with high levels of soluble sugars, cellulose, and hemicellulose, low levels of lignin and ash, and high biomass digestibility [13–15]. Grey relational grade analysis (GRA), which employs matrix calculation to quantify data at different relational levels and transforms soluble sugars, cellulose, hemicellulose, lignin, and ash contents and biomass digestibility into a single grey relational grade [16], could permit JA stem feedstock quality to be comprehensively and fairly evaluated by assigning a unique feedstock quality score (FQS). In order to meet the demands of industry, the FQS values of JA were further assigned to different feedstock quality grades (FQG).

High-throughput methodologies are often necessary for screening large numbers of lignocellulosic biomass samples [17]. Recently, NIRS coupled with multivariate calibrations such as partial least squares regression (PLSR) and partial least squares-discriminant analysis (PLS-DA) has rapidly become a key method for addressing this problem [17–21]. As the most common analytical approach, PLS has been widely demonstrated in quantitative analysis to obtain accurate and reliable results comparable to wet chemical methods [17, 22]. On the other hand, since the difference in physical and chemical properties between different raw materials has great influence on the conversion performance of biofuels, it is important to distinguish different plant varieties [23]. In this sector, several attempts have been made to address this problem [24]. So far, however, there has been little discussion about the application of NIRS in JA biomass for quantitative prediction of chemical composition and biomass digestibility and qualitative analysis of germplasm resources [17, 23, 25]. While it is universally accepted that PLS cannot completely solve data over-fitting of NIRS data, a serious hindrance to the application and promotion of this technology, variable selection techniques have subsequently emerged as powerful platforms for addressing this issue [26]. Meanwhile, a recent advance in molecular spectroscopy known as model population analysis (MPA) has been demonstrated to overcome this problem [27, 28]. As the most famous MPA algorithm, variable selection using competitive adaptive reweighted sampling (CARS), allows for selection of an optimal variable subset existing within the full spectra that can be coupled to PLSR to generate a model based on the simple, but effective “survival of the fittest” principle of Darwin’s Evolution Theory [29]. In addition, the random frog (RF) method, originally proposed for gene selection and disease classification, has recently become an efficient reversible jump Markov Chain Monte Carlo-like approach that further enhances variable selection [30]. Therefore, it is essential to evaluate the MPA-optimized PLSR or MPA-optimized PLS-DA models for use in high-throughput qualitative and quantitative analysis of JA stem biomass.

In this study, we conducted a series analysis of 59 representative JA clones collected from six regions of China for chemical composition, biomass digestibility, and NIRS results. Based on the reliable physical and chemical data, the MPA-optimized PLSR models were developed for rapidly and precisely predicting the chemical composition and biomass digestibility of JA clones. To obtain ideal bioethanol feedstocks, GRA model was performed to comprehensively evaluate JA stem samples by FQS so that the nationwide JA clones could be fairly assessed and assigned to different FQGs. Finally, the MPA-optimized PLS-DA models are developed for rapid and accurate classification of FQGs across the nationwide JA population.

## Results

### Variations of chemical composition and biomass digestibility of JA accessions for NIRS

**Fig. 1:**
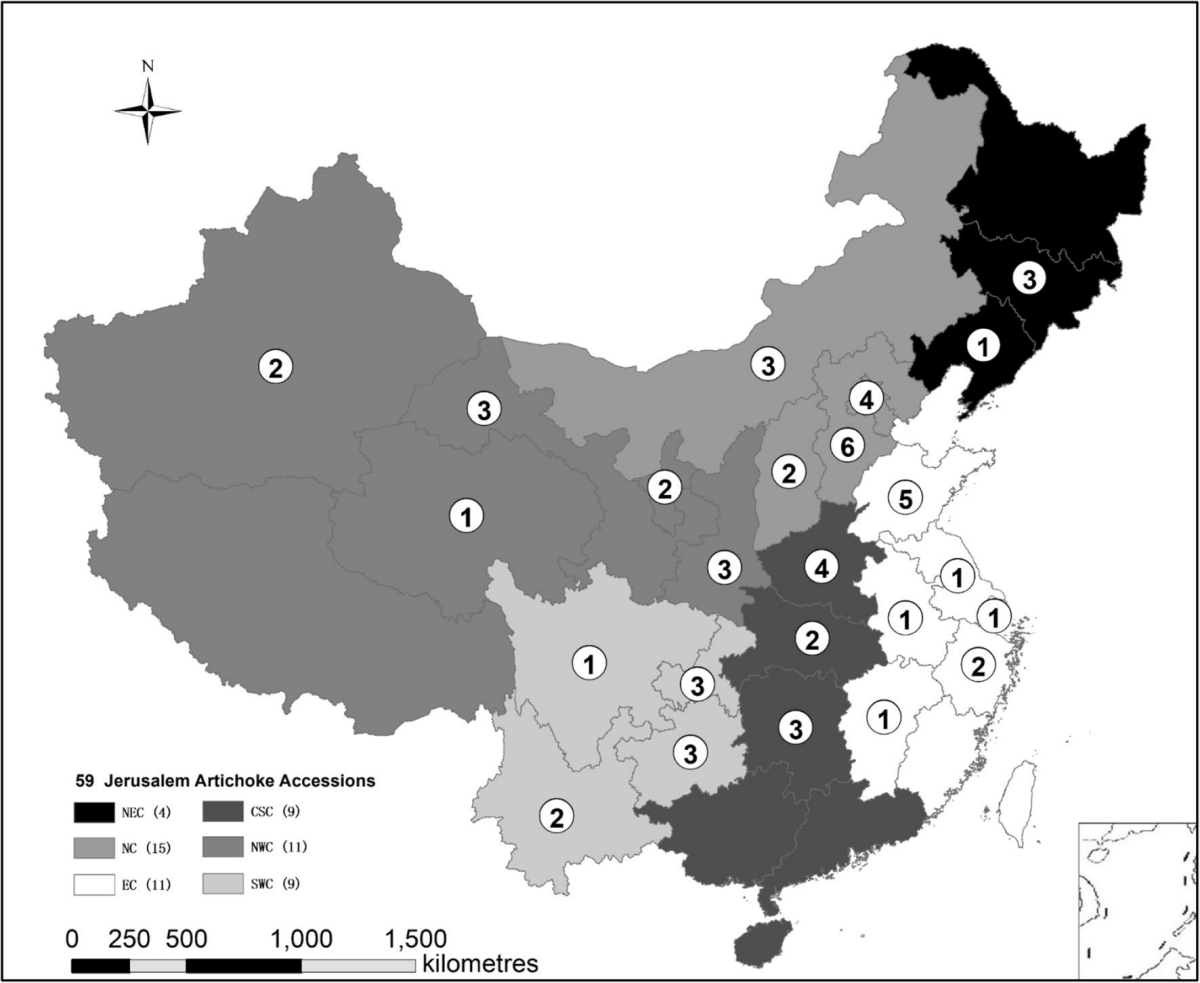
Spatial distribution of 59 Jerusalem artichoke accessions selected from typical growth regions. *NEC* Northeast China, *NC* North China, *EC* East China, *CSC* Central-South China, *NWC* Northwest China, and *SWC* Southwest China. The number in a circle (e.g., ➀) represents the number of Jerusalem artichoke accessions collected in that province

In 2006, after JA was characterized as a potential bioenergy crop, the National Energy R&D Center for Non-food Biomass initiated a long-term project for nationwide JA germplasm resources collection and identification [7–9, 11]. As a result, a total of 59 representative JA accessions were selected from 24 provinces with distinctive environments and climates (Fig. 1). According to the National Bureau of Statistics of China, these geographic areas could be divided into six regions including Northeast China (NEC), North China (NC), East China (EC), Central-South China (CSC), Northwest China (NWC), and Southwest China (SWC) [1]. Due to their distinct geographic locations and variable genotypes, these JA clones exhibited remarkable variations either in phenotype or in yield performance, demonstrating high potential for use in bioenergy crop genetic modification or high-quality biomass feedstock selection [7–9, 31]. Therefore, these JA accessions could be deemed an ideal sample population for the analysis of cell wall chemical and physical characteristics.

**Fig. 2:**
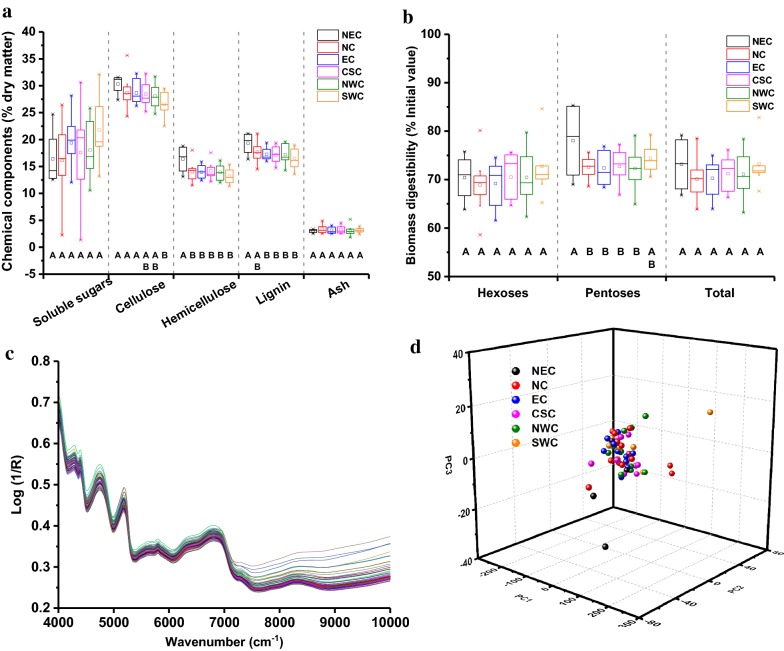
Diversity of chemical and physical properties of 59 Jerusalem artichoke accessions from six typical regions. **a** Chemical component. **b** Biomass digestibility. **c** Original near-infrared spectra. **d** Scores distribution of principal component analysis. For levels of each analyzed quality specification among the JA samples, capital letters indicate significant differences among six typical regions at *P* < 0.001. Dry matter is the dry Jerusalem artichoke sample before soluble sugar extraction. Log(1/*R*) is used to relate near-infrared reflectance to concentration of absorbing material. *NEC* Northeast China, *NC* North China, *EC* East China, *CSC* Central-South China, *NWC* Northwest China, and *SWC* Southwest China

Sample diversity was clearly demonstrated by the varying levels of soluble sugars, cellulose, hemicellulose, lignin, and ash in JA stem samples (Fig. 2a). On the one hand, it was obvious that JA stem contains a relatively high level of soluble sugars, cellulose, and hemicellulose, with mean values of 18.2%, 28.3%, and 14.0%, respectively. Among JA samples from all investigated regions, those from SWC displayed significantly lower cellulose content (22.5–29.5%), while significant higher hemicellulose level was found in JA samples from NEC (13.1–18.4%) (*P* < 0.001). On the other hand, both ash and lignin levels in JA stem were relatively lower than those in other bioenergy feedstocks [17], although JA samples from NEC contained a significantly higher lignin level than did JA samples from other regions (*P* < 0.001). Notably, the JA population studied here exhibited relatively higher levels of fermentable carbohydrates with lower levels of lignin and ash, indicating a relatively lower biomass recalcitrance than that observed for other feedstocks [15, 31]. In this study, biomass digestibility was defined by accounting for hexoses, pentoses, and total carbohydrates generated from soluble sugars-extracted JA samples after a combined alkali-based pretreatment and saccharification assay [32]. As expected, a large variation of biomass digestibility was observed among the sample population (Fig. 2b). Specifically, yields of hexoses ranged from 58.6 to 84.6% after pretreatment and subsequent enzymatic hydrolysis, while yields of pentoses ranged from 64.9 to 85.3%, leading to yields of total carbohydrates ranging from 63.2 to 82.8% among assayed JA clones. Meanwhile, ANOVA analysis demonstrated that there were no significant differences in biomass digestibility among JA clones collected from various regions, except for the yield of pentose released from JA clones from NEC. Hence, this extensive collection of JA samples exhibits high levels of desired components and biomass digestibility, indicating that JA stem could be considered as an ideal feedstock for biofuel production.

**Fig. 3:**
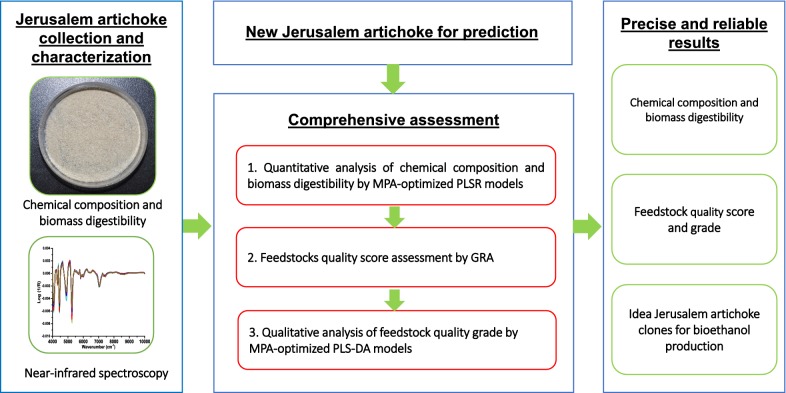
Flowchart of the comprehensive assessment for ideal Jerusalem artichoke bioethanol feedstock. *MPA* model population analysis, *PLSR* partial least squares regression, *PLS*-*DA* partial least squares-discriminant analysis, *GRA* grey relational grade analysis

NIRS, recorded from 10,000 to 4000 cm^−1^ with a resolution of 4 cm^−1^, displayed high absorption intensity with obvious baseline discrepancies and reflectance peak shifts (Fig. 2c). It is well known that the strong peaks observed in different spectral regions are attributable to frequency doubling and frequency combining characteristics of vibrations of hydrogen-containing molecules [24, 33]. Thus, the corresponding intensity of these peaks could be used to obtain information regarding biological characteristics, physical structure, and chemical composition of biomass [24, 25]. In general, the main absorption band peaks occurred within the range from 4000 to 7500 cm^−1^, demonstrating species-level similarity among JA clones. In this study, principal component analysis (PCA) was further developed for sample comparison, as well as to identify outliers. The PCA relies on projecting spectral variables on several reconstructed variables which are representative of original NIRS variation [34]. As shown in Fig. 2d, the NIRS plots of the 59 JA clones displayed a uniformly mixed and symmetrical distribution with respect to the three principal components (accounting for 93.3% of spectral variance), indicating that JA samples from six typical regions may have variable cell wall chemical and physical characteristics. Consequently, it was shown that the 59 sampled JA populations displayed relatively diverse cell wall chemical and physical characteristics, all of which would be suitable for NIRS modeling. To clearly explain the comprehensive assessment, a brief overview of is available in Fig. 3.

### Optimization of spectral variable selection and samples sets partitioning

**Fig. 4:**
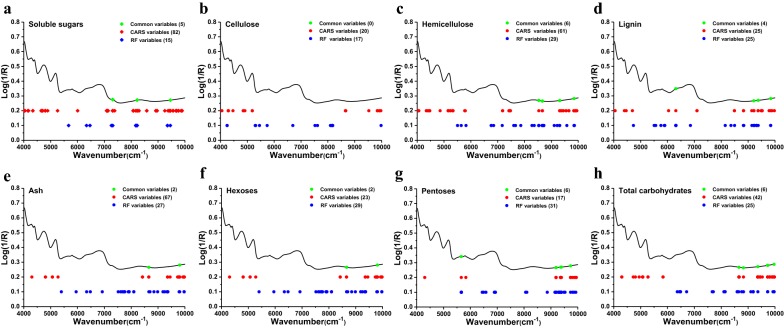
The optimal variable selection in calibration subsets by CARS and RF. **a** Soluble sugars. **b** Cellulose. **c** Hemicellulose. **d** Lignin. **e** Ash. **f** Hexoses released after pretreatment followed by enzymatic hydrolysis. **g** Pentoses released after pretreatment followed by enzymatic hydrolysis. **h** Total carbohydrates released after pretreatment followed by enzymatic hydrolysis. Log(1/*R*) is used to relate near-infrared reflectance to concentration of absorbing material. *CARS* competitive adaptive reweighted sampling, *RF* random frog

Judicious selection of spectral information is a crucial step for successful NIRS modeling, which not only permits the collection of strong informative variables but also removes interference due to uninformative variables [23, 26]. In this study, two MPA algorithms including CARS and RF were employed for spectral variable selection of chemical components and biomass digestibility of JA clones. Obviously different spectral variable sets were generated using these two MPA algorithms, which closely reflected their very different theoretical underpinnings (Fig. 4). In general, the populations of spectral variable sets selected by CARS were generally larger than those selected by RF, except for hexoses and pentoses sets. It has been reported that the strong peak at approximately 5150–5195 cm^−1^, a peak primarily attributed to O–H asymmetric stretching and O–H deformation bands of water, was believed to interfere with the prediction of other bond species [17, 23]. Interestingly, both CARS and RF successfully avoided the selection of variables from this spectral region in this study. This result could be due to that most absorption bands of cell wall polymers were correlated with the vibrations from O–H groups, which could be strongly interfered by the absorption of water and further lead to poorer correlationship between the discussed spectral region and cell wall polymers [22, 23, 34]. For the prediction of soluble sugars, cellulose, and hemicellulose, the most important spectral regions were identified at 4015–4022, 4285–4296, 4392–4412, 4760–4780, 5776–5796, 6329–6336, 6775–6822, and 7305–7328 cm^−1^ [24, 34, 35]. Meanwhile, lignin was identified at 4015–4022, 4392–4412, and 5776–5796 cm^−1^ according to the stretching vibration (O–H, C–H, C–O, and C–C) and the overtone stretching band of O–H [22, 23]. Notably, the MPA algorithms that were employed exhibited significant efficiency in variable selection within the aforementioned spectral regions (Fig. 4a–d). As previously reported, ash and biomass digestibility could be indirectly predicted by identifying the types of adjacent organic bonds within the biomass sample. Nevertheless, very few NIRS studies have been used to identify specific spectral regions that were predictive for this characteristic [17, 23, 25]. In the present study, very different spectral variable sets were successfully obtained using these two MPA algorithms for prediction of ash and biomass digestibility (Fig. 4e–h). Although they made up only a small proportion of the full spectra consisting of 1557 variables, these selected variables could effectively reduce the high collinearity of NIRS to overcome the defect of PLS [29].

It is well known that strong multivariate calibration relies heavily on both representative calibration subsets and external validation subsets [23]. In this study, the Kennard–Stone (KS) algorithm was employed for sample subsets partitioning due to its past frequent use for this purpose. For accurate and robust multivariate calibration, one of every five samples was included in the validation subset based on full spectra and two kinds of characteristic spectra, while the remaining samples were used for the calibration subset. As shown in Additional file 1: Fig. S1, the solid lines and dashed lines superimposed upon each histogram represent normal distributions that were used to delineate the discrepancy between each histogram and normality. The broad range of values for each specification could largely be attributed to the ranges in JA native spatial geographical distribution and genotypes. In general, for calibration and validation of chemical components and biomass digestibility of 59 JA accessions, histograms based on full spectra, CARS-optimized spectra, and RF-optimized spectra displayed relatively broad and approximately normal distributions. Moreover, most calibration subsets showed nearly the same distributions as the corresponding validation subsets, whereas all lacked distinct bimodal, skewed, or uniform distributions. Meanwhile, similar results were found in principal component plots distributions based on full spectra, CARS-optimized spectra, and RF-optimized spectra (Additional file 1: Fig. S2) and the 3D score plots of NIRS data from both calibration and validation subsets were well mixed and displayed relatively symmetrical distributions. Therefore, KS algorithms facilitated the optimization of both the calibration subset and related validation subset, making them suitable for subsequent multivariate calibrations.

### PLSR modeling for chemical composition

**Fig. 5:**
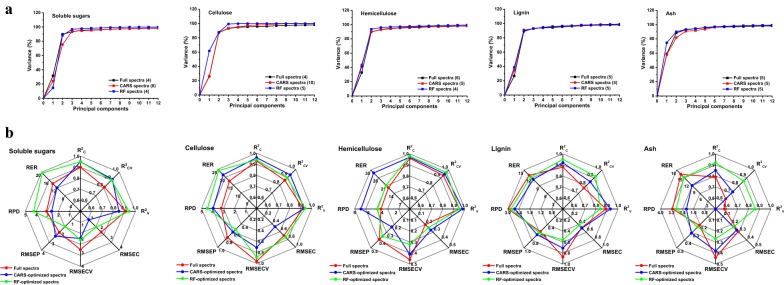
The characterization of PLSR models for chemical composition. **a** Principal components selection of PLSR models for explaining the NIRS variance. **b** Predictive performance of PLSR models for chemical composition. *CARS* competitive adaptive reweighted sampling, *RF* random frog, *R*^*2*^_*C*_ coefficient determination of calibration, *R*^*2*^_*CV*_ coefficient determination of cross-validations, *R*^*2*^_*V*_ coefficient determination of validations, *RMSEC* root mean standard error of calibration, *RMSECV* root mean standard error of cross-validation, *RMSEP* root mean square error of prediction, *RPD* the ratio of performance to deviation, *RER* the range error ratio

Based on the optimized spectra and sample subsets, 10 MPA-optimized PLSR models were developed for soluble sugars, cellulose, hemicellulose, lignin, and ash. In order to conduct a fair comparison, five PLSR models were developed using full spectra and served as controls. During the developmental process, the numbers of samples for calibration and validation subsets were further reduced by the removal of sample outliers using Chauvenet’s criterion [35]. To enhance the robustness of multivariate calibrations, all PLSR models were fully cross-validated using the “leave-one-out” method, where a single observation selected from calibration sample subsets was used as the validation data for each cross-validated process [17, 23]. The optimal number of principal components (PCs) for each model was determined using root mean standard error of calibration (RMSEC) and the root mean standard error of cross-validation (RMSECV). In this case, 4 to 10 optimal PCs were obtained for 15 PLSR models accounting for over 96% of the variance, which further reduced the danger of over-fitting (Fig. 5a). Interestingly, the PCs of RF-optimized PLSR models generally accounted for higher values of variance than did CARS-optimized PLSR models and controls.

Summary statistics for the PLSR calibrations of chemical composition are provided in Table 1. In general, all the chemical components were successfully predicted by PLSR models and their uncertainties approximated those observed for wet chemical measurements, as indicated by low RMSEC (0.20–2.14) and RMSECV (0.27–2.78) values obtained. As a result, the relative high coefficients of determination of calibration (*R*^2^_C_) and cross-validations (*R*^2^_CV_) were obtained within the ranges of 0.79–0.98 and 0.63–0.97, respectively. Significantly, both types of MPA-optimized PLSR calibration models generally displayed higher stability than did the controls, which exhibited relatively low RMSEC and RMSECV and high *R*^2^_C_ and *R*^2^_CV_. Consequently, these results indicate that MPA could significantly improve the robustness of PLSR calibrations.

**Table 1:** Summary statistics for PLSR calibration models for chemical components

Parameter	*N*	Full spectra				CARS-optimized spectra				RF-optimized spectra			
		RMSEC	*R* ^2^_C_	RMSECV	*R* ^2^_CV_	RMSEC	*R* ^2^_C_	RMSECV	*R* ^2^_CV_	RMSEC	*R* ^2^_C_	RMSECV	*R* ^2^_CV_
Soluble sugars	40	2.14	0.90	2.78	0.81	0.88	0.95	1.98	0.91	1.63	0.95	2.07	0.90
Cellulose	45	0.70	0.93	0.96	0.86	0.47	0.96	0.71	0.93	0.74	0.94	0.89	0.89
Hemicellulose	45	0.20	0.96	0.46	0.93	0.27	0.97	0.41	0.95	0.24	0.98	0.32	0.97
Lignin	45	0.48	0.88	0.87	0.77	0.49	0.92	0.71	0.85	0.42	0.95	0.56	0.92
Ash	45	0.29	0.79	0.44	0.63	0.27	0.85	0.38	0.72	0.23	0.92	0.27	0.86

*N* number of samples, *CARS* competitive adaptive reweighted sampling, *RF* random frog, *RMSEC* root mean standard error of calibration, *R*^*2*^_*C*_ coefficient determination of calibration, *RMSECV* root mean standard error of cross-validation, *R*^*2*^_*CV*_ coefficient determination of cross-validation

In the present study, fair prediction using externally validated samples was conducted to evaluate calibration equations. Summary statistics for the prediction of soluble sugars, cellulose, hemicellulose, lignin, and ash are provided in Table 2. In general, RF-optimized PLSR models performed better or similar as CARS-optimized models and controls when comparing values of the root mean square error of prediction (RMSEP) and *R*^2^_V_ for prediction of soluble sugars, cellulose, and lignin. On the contrary, CARS optimization could significantly improve the performance of PLSR multivariate calibrations for hemicellulose prediction. In recent years, numerous studies have reported that ash level is not easily or directly measurable by NIRS, due to its inorganic nature [17, 18, 24, 36]. Similarly, the statistics of CARS-optimized PLSR models and controls suggest fair to the poor prediction for ash in the present study, rendering these two methods suitable only for very rough screening. However, inorganic ash was successfully predicted by RF-optimized PLSR models with significantly higher *R*^2^_V_ and lower RMSEP.

**Table 2:** Summary statistics for external validation of PLSR calibration models for chemical components

Parameter	*N*	Full spectra				CARS-optimized spectra				RF-optimized spectra			
		RMSEP	*R* ^2^_V_	RPD	RER	RMSEP	*R* ^2^_V_	RPD	RER	RMSEP	*R* ^2^_V_	RPD	RER
Soluble sugars	13	2.17	0.91	3.07	14.18	2.55	0.85	2.61	12.07	1.57	0.94	4.24	19.60
Cellulose	14	0.75	0.92	3.21	17.49	0.61	0.93	3.95	21.51	0.54	0.94	4.46	24.29
Hemicellulose	14	0.39	0.95	4.18	19.12	0.23	0.98	7.08	32.42	0.35	0.95	4.65	21.31
Lignin	14	0.58	0.89	2.98	12.97	0.66	0.93	2.61	11.40	0.61	0.84	2.83	12.33
Ash	14	0.21	0.58	3.13	16.05	0.31	0.08	2.12	10.87	0.23	0.86	2.86	14.65

*N* number of samples, *CARS* competitive adaptive reweighted sampling, *RF* random frog, *RMSEP* root mean square error of prediction, *R*^*2*^_*V*_ coefficient determination of validation, *RPD* the ratio of performance to deviation, *RER* the range error ratio

Recent studies suggest that for research calibrations, excellent calibration models must exhibit a ratio of performance to deviation (RPD) and range error ratio (RER) values greater than 3 and 15, respectively [22–25]. In this study, almost all RPD and RER values of RF-optimized PLSR models were higher or similar to standard values (Table 2), resulting in relatively better correlations between predicted and reference values for chemical components (Additional file 1: Fig. S3). In order to achieve greater visual comprehension, the spider diagram was adopted for comparing the predictive performance of employed PLSR models (Fig. 5b). It was obvious that RF was a more efficient algorithm than CARS for improving the predictive performance of PLS multivariate calibrations. Taken together, these results indicate that MPA optimization shows promise for improving the accuracy and robustness of PLS multivariate calibrations and that RF-optimized PLSR models are adequate for the determination of JA chemical composition.

### NIRS modeling for biomass digestibility

**Fig. 6:**
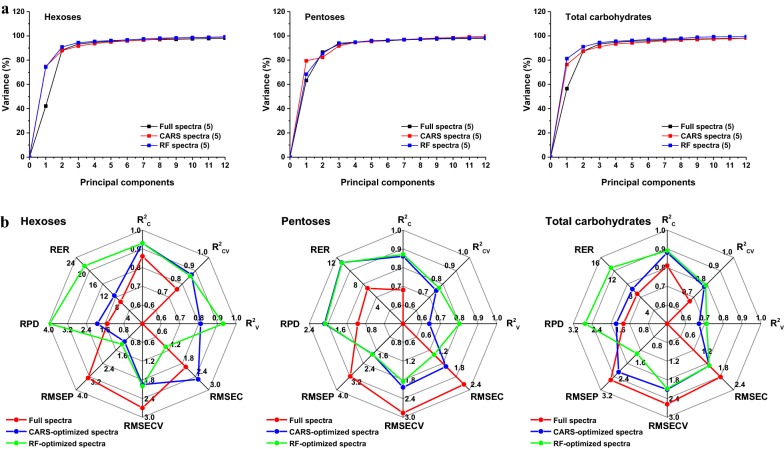
The characterization of PLSR models for biomass digestibility. **a** Principal components selection of PLSR models for explaining the NIRS variance. **b** Predictive performance of PLSR models for biomass digestibility. *CARS* competitive adaptive reweighted sampling, *RF* random frog, *R*^*2*^_*C*_ coefficient determination of calibration, *R*^*2*^_*CV*_ coefficient determination of cross-validations, *R*^*2*^_*V*_ coefficient determination of validations, *RMSEC* root mean standard error of calibration, *RMSECV* root mean standard error of cross-validation, *RMSEP* root mean square error of prediction, *RPD* the ratio of performance to deviation, *RER* the range error ratio

To assess biomass digestibility, six similarly optimized MPA-optimized PLSR models were developed for JA accessions obtained nationwide, while three PLSR models served as controls. Similarly, RF-optimized PLSR models generally produced higher values of variance than did CARS-optimized PLSR models and controls at optimal *PCs* (Fig. 6a). Table 3 provides an overview of nine PLSR calibrations for the prediction of hexoses, pentoses, and total carbohydrates. In general, the prediction models of biomass digestibility exhibited a slight decrease in *R*^2^_C_ values (0.68–0.93) and *R*^2^_CV_ values (0.48–0.87) than did prediction models of chemical composition. However, six MPA-optimized PLSR models for biomass digestibility showed significantly higher *R*^2^_C_ (0.86–0.93) and *R*^2^_CV_ (0.75–0.87) values than corresponding controls. Meanwhile, extremely low RMSEC (1.05–1.55) and RMSECV (1.85–2.11) values were also obtained using MPA-optimized calibrations, indicating superior stability.

**Table 3:** Summary statistics for PLSR calibration models for biomass digestibility

Parameter	*N*	Full spectra				CARS-optimized spectra				RF-optimized spectra			
		RMSEC	*R* ^2^_C_	RMSECV	*R* ^2^_CV_	RMSEC	*R* ^2^_C_	RMSECV	*R* ^2^_CV_	RMSEC	*R* ^2^_C_	RMSECV	*R* ^2^_CV_
Hexoses	45	1.96	0.86	2.70	0.76	1.29	0.93	1.97	0.87	1.05	0.93	2.00	0.86
Pentoses	45	2.20	0.68	2.86	0.48	1.55	0.86	2.04	0.75	1.12	0.87	1.85	0.77
Total	45	1.93	0.81	2.58	0.67	1.52	0.88	2.11	0.78	1.52	0.89	2.08	0.79

*N* number of samples, *CARS* competitive adaptive reweighted sampling, *RF* random frog, *RMSEC* root mean standard error of calibration, *R*^*2*^_*C*_ coefficient determination of calibration, *RMSECV* root mean standard error of cross-validation, *R*^*2*^_*CV*_ coefficient determination of cross-validation

Subsequently, lower RMSEP (1.24–2.55), higher RPD (1.74–3.96), and RER (8.38–20.96) values were obtained using robust MPA-optimized calibrations (Table 4). In particular, the 14 externally validated samples exhibited strong correlations between predicted and reference values (Additional file 1: Fig. S3B, C). By contrast, the controls demonstrated very poor predictive capacity (Table 4 and Additional file 1: Fig. S3A). These results demonstrate that MPA could clearly improve upon prediction performance accuracies of the PLSR model for biomass digestibility prediction, which verified our previously stated results that CARS and RF displayed superior efficiency for the selection of both informative variables and elimination of uninformative variables (Fig. 4). Notably, comparison of MPA algorithms indicated that RF was more efficient than CARS in improving the performance of PLSR calibrations for predicting yields of hexoses, pentoses, and total carbohydrates released after pretreatment followed by enzymatic hydrolysis (Fig. 6b). Together these results provide important insights into how the optimization of spectral variable selection by MPA could significantly enhance both the stability and accuracy of PLSR models for chemical composition and biomass digestibility. Such models form the basis of a precise and efficient methodology for predicting both chemical composition and biomass digestibility of JA feedstock.

**Table 4:** Summary statistics for external validation of PLSR calibration models for biomass digestibility

Parameter	*N*	Full spectra				CARS-optimized spectra				RF-optimized spectra			
		RMSEP	*R* ^2^_V_	RPD	RER	RMSEP	*R* ^2^_V_	RPD	RER	RMSEP	*R* ^2^_V_	RPD	RER
Hexoses	14	3.28	0.44	1.50	7.92	2.55	0.81	1.93	10.19	1.24	0.93	3.96	20.96
Pentoses	14	3.18	0.15	1.16	6.42	1.84	0.64	2.01	11.09	1.85	0.80	1.99	11.03
Total	14	2.72	0.35	1.49	7.21	2.34	0.67	1.74	8.38	1.45	0.71	2.80	13.52

*N* number of samples, *CARS* competitive adaptive reweighted sampling, *RF* random frog, *RMSEP* root mean square error of prediction, *R*^2^_*V*_ coefficient determination of validation, *RPD* the ratio of performance to deviation, *RER* the range error ratio

### Comprehensive assessment of feedstock quality score

**Fig. 7:**
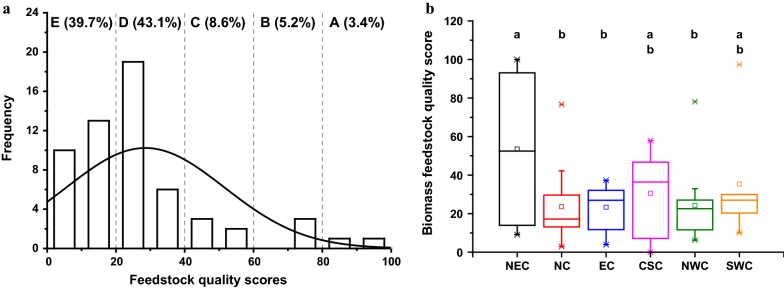
Feedstock quality grades distribution of 59 Jerusalem artichoke accessions in China. **a** National scope. **b** Regional scope. Different small letters indicate significant differences among six typical regions at *P* < 0.001. The solid lines overlaid upon each histogram represent normal distributions and were used to embody the discrepancy between histogram and normality. *NEC* Northeast China, *NC* North China, *EC* East China, *CSC* Central-South China, *NWC* Northwest China, and *SWC* Southwest China

Based on chemical composition and biomass digestibility (total carbohydrates released after pretreatment and subsequent enzymatic hydrolysis), the feedstock quality of tested JA accessions was comprehensively evaluated using the GRA model. As shown in Fig. 7a, solid lines represent the normal distribution and are intended to highlight any discrepancy between the histogram and normality. With regard to FQS, frequency refers to the number of samples within a given range and the percentage of each FQG is indicated above the histograms. In general, the FQS distribution was similar to a normal distribution, which is not unexpected for a nationwide feedstock population. Moreover, the mean value of FQS was 28.6 and majority of the samples fell within D (43.1%) and E (39.7%) grades. By contrast, only 3.4% and 5.2% of JA samples were assigned to A and B grades (details could be found in Additional file 1: Table S1). Hence, there is a growing need to focus efforts on JA germplasm resource selection, breeding, and genetic modification to improve biomass feedstock quality for efficient bioethanol production.

Notably, the JA accessions obtained from six areas of China displayed very diverse FQS distributions (Fig. 7b) and mean FQS values were ranked in the order of NEC (53.5) > SWC (35.3) > CSC (30.1) > NWC (24.3) > NC (23.6) > EC (23.3). ANOVA analysis showed that JA accessions from NEC exhibited significantly higher FQS than those from NC, EC, and NWC (*P* < 0.001). However, high-quality JA accessions could be found in most sampled regions (except for EC). Summary statistics of chemical components, biomass digestibility, and FQS of JA accessions assigned to grade A and B are detailed in Table 5. As expected, each selected JA accession possessed a high level of soluble sugars, cellulose, and hemicellulose, low levels of lignin and ash, and outstandingly high biomass digestibility. Therefore, these accessions are currently the most ideal JA feedstocks for bioethanol production in China, demonstrating that the GRA was adequate for high-throughput biomass feedstock quality evaluation.

**Table 5:** The statistics of Jerusalem artichoke accessions ideal for bioethanol production in China

Parameter	E025	E047	E026	E058	E053	E044	E059
Soluble sugars (% dry matter)	15.4	32.1	13.1	23.4	26.4	30.1	21.8
Cellulose (% dry matter)	30.9	22.5	31.6	24.8	24.3	23.2	27.5
Hemicellulose (% dry matter)	18.4	11.4	18.8	12.0	11.5	11.4	13.4
Lignin (% dry matter)	21.1	13.6	20.9	14.3	14.5	14.2	14.8
Ash (% dry matter)	3.4	3.7	3.0	5.2	4.9	3.9	4.5
Biomass digestibility (% total carbohydrates)	79.2	82.8	77.3	78.4	78.5	78.8	76.1
Region	NEC	SWC	NEC	NWC	NC	SWC	CSC
Feedstock quality score	100.0	97.4	86.2	78.1	76.7	70.7	60.1
Feedstock quality grade	A	A	A	B	B	B	B

*NEC* northeast China, *SWC* southwest China, *NWC* northwest China, *NC* north China, *CSC* central-south China

### Qualitative analysis of feedstock quality grade

For the purposes of the industrial application, further research should focus on rapid and precise classification of a large number of biomass feedstocks into different FQGs to achieve commoditization of lignocellulosic biofuel. In this work, KS algorithm-partitioned sample subsets coupled to both CARS and RF algorithms were employed to develop two optimized PLS-DA models for FQG classification of JA stem biomass, while PLS-DA models based on full spectra served as controls. Summary statistics of three PLS-DA models for qualitative analysis of FQG are presented in Table 6. Notably, the FQG values of JA stem were successfully classified using PLS-DA models to obtain relatively higher values of the multiple coefficients of determination (*R*^2^: 0.76–0.96) and the explained variation in the test set (*Q*^2^: 0.52–0.83). In addition, it was obvious that two MPA algorithms could significantly enhance the stability of PLS-DA models. Regarding classification accuracy, the highest correct rates, 97.8% in the calibration subset and 91.1% in the validation subset, were obtained using the robust RF-optimized PLS-DA model, which were superior to rates obtained using the CARS-optimized PLS-DA model. Therefore, the RF algorithm was more efficient than the CARS algorithm for improving the predictive performance of PLS multivariate calibrations. Overall, these results indicate that the optimization of spectral variable selection by MPA could significantly enhance the stability and classification accuracy of PLS-DA multivariate calibrations, consistent with the quantitative determinations of JA stem chemical composition and biomass digestibility.

**Table 6:** Summary statistics of PLS-DA models for qualitative analysis of biomass feedstock quality grades

Parameter	PCs	*R* ^2^	*Q* ^2^	Calibration		Validation	
				*N*	Correct (%)	*N*	Correct (%)
Full spectra	4	0.76	0.52	45	97.8	14	86.7
CARS-optimized spectra	7	0.96	0.83	45	95.6	14	88.9
RF-optimized spectra	3	0.91	0.80	45	97.8	14	91.1

*PCs* principal components, *R*^2^ the multiple coefficients of determination, *Q*^2^ the explained variation in the test set, *N* number of samples

## Discussion

Evaluation and selection of ideal feedstock among bioenergy crops are necessary to enhance lignocellulosic biofuel production [37, 38]. Recent developments in biochemical conversion have demonstrated that the chemical and physical properties of lignocellulose are crucial indicators of biomass feedstock quality [12]. In terms of chemical composition, soluble sugars, cellulose, and hemicellulose act as primary resources for biofuel production, while lignin and ash are considered useless components or barriers to biochemical conversion [39]. Therefore, inherent variability in chemical composition fundamentally determines the theoretical energy potential of lignocellulosic feedstock. With respect to physical structure, lignocellulose is a compact dimensional molecule, which gives rise to biomass recalcitrance such that deconstruction of any of physical properties is constrained by the other properties. Recently, results of a combined pretreatment and saccharification assay have increased our understanding of phenomena that include “biomass reactivity” or “biomass digestibility,” properties which could be used to directly predict usability of lignocellulosic feedstock [32]. Therefore, the current knowledge suggests that chemical composition and biomass digestibility should be considered together during lignocellulosic biomass evaluation and selection. Prior to this study, few reports have described the comprehensive assessment of lignocellulosic biomass based on both chemical composition and biomass digestibility [32, 37, 38, 40, 41]. In addition, due to innate variability in chemical composition and biomass digestibility, it is critical to understand that not all lignocellulosic biomass is suitable for conversion into biofuels and biochemicals [14]. Therefore, particular attention should be paid to ideal feedstock selection when JA stem biomass was concerned.

In the current study, we determined the variability in chemical composition and biomass digestibility of sugar-rich stem biomass of 59 JA clones collected nationwide. These JA accessions were uniformly distributed within longitudes between 82.06 and 126.75 and latitudes between 25.60 and 44.86 and exhibited diverse genotypes and phenotypes [7–9]. Notably, innate JA feedstock quality variability based on chemical composition and biomass digestibility was identified at both national and regional levels (Fig. 2), which could be caused by the interaction between diverse JA genotypes and their various environments. These results suggest that a potential exists for selection of ideal varieties to enhance biofuel conversion or bioenergy crop breeding and genetic modification. Besides, ideal bioethanol feedstock could be identified by high levels of carbohydrates (soluble sugars, cellulose, and hemicellulose), high biomass digestibility and low level of waste components (lignin and ash) [14, 17]. For the purpose of screening the ideal bioethanol feedstock, we transformed the feedstock quality properties to a fair FQS and further classified it into five FQG levels. As a result, 7 clones from 6 sampled regions were deemed superior biomass feedstocks for bioethanol production. Clearly, these results demonstrate the high potential of JA to serve as an ideal bioenergy crop for nationwide cultivation. Although the JA accessions in this study were highly representative, these results should be interpreted with caution due to the limited size of the sample population. Therefore, attention should be paid to the continued collection and identification of germplasm resources.

With the development of computational science, NIRS coupled with PLS has gained extensive popularity for the qualitative and quantitative analysis of chemical and physical properties of biomass feedstock due to its high-throughput, low-cost, and non-destructive nature [17, 19, 20, 22–24, 33, 36]. However, the intrinsically disadvantage of PLS was that it requires a crucial process to build a credible model for given chemical or biological data, which is known as spectral variable selection [23]. In this sector, MPA has offered great advantages by statistically analyzing the distribution of an interested outcome of the sub-models derived with the aid of Monte Carlo Sampling, which could significantly improve the accuracy and robustness of prediction models [26, 28]. In this study, two novel MPA algorithms based on different theory (CARS and RF) were employed to establish a series of optimal spectral variable subsets derived from full NIRS data. One interesting finding was that only 17–82 and 15–31 spectral variables were selected by CARS and RF from 1557 variables within full spectra, respectively. This finding indicates that MPA could judiciously identify most of the informative spectral variables while eliminating the uninformative spectral variables [23, 29, 30]. Another important finding was that several variables were collectively selected by both of these algorithms for each predictive indictor, providing very important information that could be used for further improve prediction of biomass chemical and physical properties [23]. Based on these optimized spectral variable subsets, a series of MPA-optimized PLSR models and MPA-optimized PLS-DA models were developed for quantitative and qualitative analysis of JA biomass feedstocks. The results indicated that RF was better for improving both qualitative and quantitative PLS models than CARS (Figs. 5, 6 and Table 6). In comparison with the NIR models of previous studies, RF-optimized PLSR models exhibited a better performance for the prediction of chemical composition (Additional file 1: Table S2) and biomass digestibility (Additional file 1: Table S3) with more reasonable values of RMSEC*, R*^2^_C_, RMSECV*, R*^2^_CV_, RMSEP*, R*^2^_V_, RPD, and RER [17, 19, 22, 23, 42]. Obviously, spectral variable selection using MPA could significantly improve the predictive performance of PLS multivariate calibration in the qualitative and quantitative analysis of biomass feedstock.

## Conclusions

In this study, 59 JA clone stems originating from six regions of China exhibited diverse chemical compositions, biomass digestibility, and variable NIRS results, which were applicable for statistical analysis and NIRS modeling. Soluble sugars, cellulose, hemicellulose, lignin, ash, and released hexoses, pentoses, and total carbohydrates were then successfully predicted via MPA-optimized PLSR models. Based on the reliable and accurate data of chemical composition and biomass digestibility, all 59 JA accessions were comprehensively evaluated for FQS by GRA and assigned into five FQGs. Notably, seven clones were identified as the most ideal JA feedstocks for bioethanol production in China. Finally, the JA accessions studied in this work were rapidly and successfully classified using a MPA-optimized PLS-DA model. In conclusion, this study provides a practical strategy of high-throughput screening lignocellulosic biomass for bioethanol production.

## Methods

### Sample collection and preparation

A total of 59 JA natural clones were collected nationwide from 2006 to 2012. JA stem samples were harvested on their dates of physiological maturity and were processed at the China Agricultural University Zhuozhou Experimental Station (39°47′ N, 115°87′ E) in Hebei Province in 2014. Stem samples were handled according to the protocols developed by the National Renewable Energy Laboratory/TP-510-42620 [43]. First, samples were ground using a crusher mill into particles 1–2 cm in size and dried at 45 °C for 48 h after heat at 105 °C for 20 min. Next, the dried particles were ground into powder and passed through a combined − 40/+ 80 mesh screen. Finally, the mesh-screened samples (as dry matter) were stored in a dry container until use. A spatial distribution map of collection sites of JA accessions was generated using ArcGIS 10.3.

### Biomass components and digestibility analysis

The main topic of this research was to build upon the laboratory-scale alkali-based conversion process developed by Li et al. [11] to develop a comprehensive and high-throughput lignocellulosic biomass feedstock screening system for bioethanol production. Soluble sugars were extracted from dried JA stem samples using distilled water and quantified using the anthrone-sulfuric acid method using a UV–VIS spectrometer (TU-1901, Beijing Purkinje General Instrument Co., Ltd., Beijing, China). A standard curve was plotted using d-glucose as the standard (Xilong Scientific Co., Ltd., China). Ash content was determined using a muffle furnace (VULCAN 3-550, Densply International, Inc., York, PA, USA) with 30 mL ceramic crucibles according to LAP NREL/TP-510-42622 [44]. Structural carbohydrates and lignin were extracted using a two-step sulfuric acid hydrolysis process with dried JA stem samples according to NREL/TP-510-42618, NREL/TP-510-42619, and NREL/TP-510-42621 with minor modifications [45–47]. Structural carbohydrates (i.e., glucose, xylose, and arabinose) were measured using an HPLC system (1260 series, Agilent Technologies, Santa Clara, CA, USA) equipped with an Aminex HPX-87H chromatography column (300 mm × 7.8 mm, particle size 9 µm, Bio-Rad Laboratories, Hercules, CA, USA). Lignin content was determined using a UV–VIS spectrometer (TU-1901, Beijing Purkinje General Instrument Co., Ltd.) and the same muffle furnace described above. Calculations of cellulose, hemicellulose, and lignin content were performed according to Li et al. [11]. Cellulose was calculated from glucose content, while hemicellulose was calculated from the sum of xylose and arabinose content values. Lignin was calculated from the sum of acid-soluble lignin and acid-insoluble lignin content values. All experiments were carried out in triplicate.

Biomass digestibility was defined by accounting for the hexoses, pentoses, and total carbohydrates released from the soluble sugars extracted biomass feedstock after alkali-based pretreatment followed by enzymatic hydrolysis [11]. For pretreatment, a 2-g quantity of soluble sugars-extracted JA sample was mixed with 40 mL 2% (w/v) sodium hydroxide and shaken at 150 rpm for 2 h at 50 °C. Next, each pretreated sample was washed five times with 20 mL distilled water and dried at 80 °C for subsequent enzymatic hydrolysis. During enzymatic hydrolysis, each pretreated sample was mixed with 0.2% (w/v) mixed-cellulases containing β-glucanase (≥ 3.6 × 10^4^ U), cellulase (≥ 360 × 10^2^ U), and xylanase ≥ 6×10^4^ U from Imperial Jade Biotechnology Co., Ltd.) in a volume of 40 mL in a 50-mL centrifuge tube and shaken at 150 rpm at 50 °C for 48 h. Glucose and xylose released after alkali-based pretreatment followed by enzymatic hydrolysis were determined by HPLC as described above. Statistical and variance (ANOVA) analysis was calculated using IBM SPSS Statistics software (ver. 24). All experiments were carried out in triplicate.

### Near-infrared spectroscopy pretreatments

JA stem dry matter was scanned and recorded in triplicate using a Thermo Antaris II FT-NIR Analyzer (Thermo Scientific, Inc., Madison, WI, USA) equipped with a diffuse reflectance accessory device. Each spectrum was averaged over 64 scans at a resolution of 4 cm^−1^ within the wavenumber range of 4000–10,000 cm^−1^ at room temperature (Additional file 2). Spectrometer control and data collection were conducted using TQ Analyst software (ver. 9.3). In order to correct spectra scatter, all spectra were first adjusted using multiplicative scatter correction. Next, the Savitzky–Golay smoothing filter and the first derivative were employed to reduce random noise and to resolve spectral peak overlap and eliminate linear baseline drift [23]. The purpose of the aforementioned corrections was to remove multiplicative and additive effects stemming from instrument settings or variations caused by the sample and environmental conditions [19]. After pretreatments, six principal component analysis models were developed using TQ Analyst software (ver. 9.3) to generate a 3D NIRS scatter plot [33].

### The development of MPA-optimized PLSR models

Based on pretreated sample spectra, both CARS and RF algorithms were applied to determine spectral variables sets using ChemDataSolution software (ver. 2.0). For a fair multivariate prediction, one of every five samples was sorted into validation sets using KS algorithms based on full spectra and two types of characteristic spectra; the remaining samples were used for generating calibration sets. Calibration and validation sets were compared in terms of both their chemical components and biomass digestibility. In addition, the NIRS plots distribution of calibration and validation sets was also compared using eighteen principal component analysis models in the present study. Based on the MPA-optimized spectra, sixteen PLSR multivariate calibrations were developed to predict soluble sugars, cellulose, hemicellulose, lignin, ash, and biomass digestibility (hexoses, pentoses, and total carbohydrates) using ChemDataSolution software (ver. 2.0); eight PLSR models based on full spectra served as controls. To select the optimum number of factors and to avoid over-fitting, the “leave-one-out” method was used for cross-validation while developing PLSR models [17, 19]. On the one hand, the robustness of PLSR models was evaluated using several chemometrics parameters, including RMSEC, RMSECV and *R*^2^_C_, and *R*^2^_CV_. On the other hand, the accuracy of each multivariate calibration model could be determined by RMSEP and *R*^2^_V_. Finally, RPD and RER were employed to ascertain MPA enhancement of prediction performance.

### Grey relational grade analysis

In this study, JA stem feedstock quality specifications could be divided into two categories: chemical components (soluble sugars, cellulose, hemicellulose, lignin, and ash) and biomass digestibility (hexoses, pentoses, and total carbohydrates). These quality specifications were used to calculate and compare the FQS values of JA samples collected from different geographic regions. To address this challenge, a high-efficiency evaluation and selection model was developed based on grey relational grade analysis theory, which is an essential component of the grey system theory formulated by Julong Deng [16]. Both chemical components and biomass digestibility (total carbohydrates released from pretreatment and subsequent enzymatic hydrolysis) were assigned the same weight of 0.5 in this study. To meet the demands of industry, the FQS values of JA samples were assigned to five grade levels and ranked in the order A (80–100) ≥ B (60–80) ≥ C (40–60) ≥ D (20–40) ≥ E (0–20). The GRA was processed using MATLAB software (ver. 2012b) and the procedure code is documented in Additional file 3.

### The development of MPA-optimized PLS-DA models

Based on full spectra and MPA-optimized spectra, three PLS-DA multivariate calibrations were developed for FQG classification using ChemDataSolution software (ver. 2.0). One of every five samples was sorted into validation sets using KS algorithms based on full spectra and two characteristic spectra and the remaining samples were used for the calibration sets. In addition, the multiple coefficients of determination (*R*^2^), the explained variation in the test set (*Q*^2^), and the correct rate were employed to ascertain MPA enhancement of classification performance [21].

## Additional files

**Additional file 1: Fig. S1.** Histograms of chemical components and biomass digestibility based on full spectra (A), CARS-optimized spectra (B), and RF-optimized spectra (C). The solid lines and dashed lines overlaid upon each histogram represent normal distributions and were used to embody the discrepancy between each histogram and normality. **Fig. S2.** PCA plots distribution of chemical components and biomass digestibility based on full spectra (A), CARS-optimized spectra (B), and RF-optimized spectra (C). **Fig. S3.** Plots of predicted versus reference values of PLSR models based on full spectra (A), CARS-optimized spectra (B), and RF-optimized spectra (C). *R*^2^_V_ represents the square of the correlation coefficients of the external validation subsets. **Table S1.** Feedstock quality grades of 59 Jerusalem artichoke accessions. **Table S2.** A summary of NIR application in different biomass feedstocks for chemical components. **Table S3.** A summary of NIR application in different biomass feedstocks for biomass digestibility.

**Additional file 2.** Raw NIRS data.

**Additional file 3.** The procedure code of grey relational grade analysis.

## Abbreviations

JA: Jerusalem artichoke
NIRS: near-infrared spectroscopy
KS: Kennard–Stone algorithm
PLSR: partial least squares regression
PLS-DA: partial least squares-discriminant analysis
PCA: principal component analysis
MPA: model population analysis
CARS: competitive adaptive reweighted sampling
RF: random frog
RMSEC: root mean standard error of calibration
RMSECV: root mean standard error of cross-validation
RMSEP: root mean square error of prediction
*R*^2^_C_: coefficient determination of calibration
*R*^2^_CV_: coefficient determination of cross-validations
*R*^2^_V_: coefficient determination of validations
RPD: the ratio of performance to deviation
RER: the range error ratio
GRA: grey relational grade analysis
FQS: feedstock quality score
FQGs: feedstock quality grades
*R*^2^: the multiple coefficient of determination
*Q*^2^: the explained variation in the test set
