# Supplementary material for: An NIRS-based assay of chemical composition and biomass digestibility for rapid selection of Jerusalem artichoke clones
Source: Biotechnol Biofuels. 2018 Dec 19;11:334. doi: 10.1186/s13068-018-1335-1 (PMC6299672; doi:10.1186/s13068-018-1335-1)
Supplement: Supplementary file 1 — Additional file 1: Fig. S1. Histograms of chemical components and biomass digestibility based on full spectra (A), CARS-optimized spectra (B), and RF-optimized spectra (C). The solid lines and dashed lines overlaid upon each histogram represent normal distributions and were used to embody the discrepancy between each histogram and normality. Fig. S2. PCA plots distribution of chemical components and biomass digestibility based on full spectra (A), CARS-optimized spectra (B), and RF-optimized spectra (C). Fig. S3. Plots of predicted versus reference values of PLSR models based on full spectra (A), CARS-optimized spectra (B), and RF-optimized spectra (C). R2V represents the square of the correlation coefficients of the external validation subsets. Table S1. Feedstock quality grades of 59 Jerusalem artichoke accessions. Table S2. A summary of NIR application in different biomass feedstocks for chemical components. Table S3. A summary of NIR application in different biomass feedstocks for biomass digestibility. [file 13068_2018_1335_MOESM1_ESM.pptx]

## Slide 1
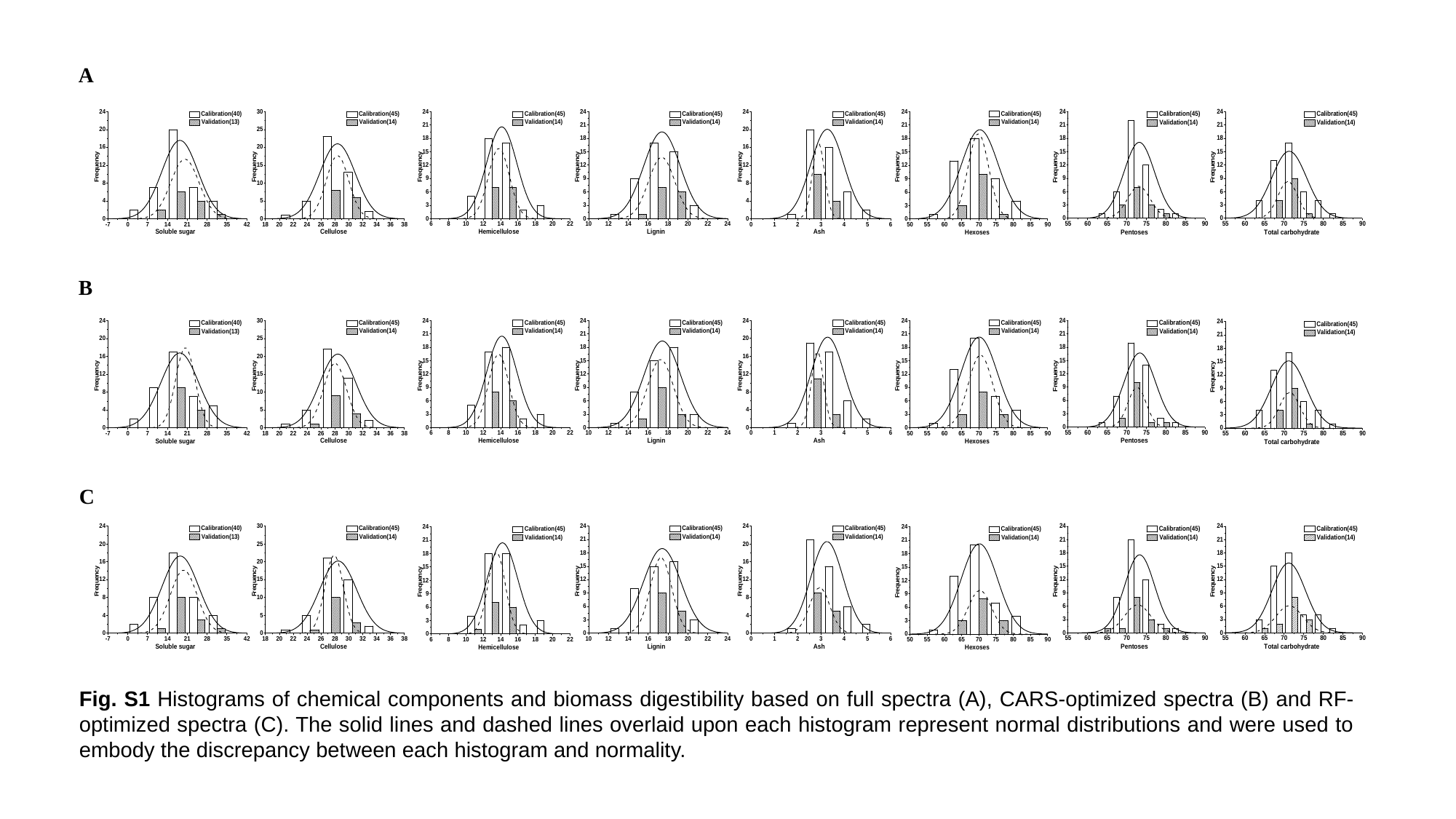

A
B
C
Fig. S1 Histograms of chemical components and biomass digestibility based on full spectra (A), CARS-optimized spectra (B) and RF-optimized spectra (C). The solid lines and dashed lines overlaid upon each histogram represent normal distributions and were used to embody the discrepancy between each histogram and normality.

## Slide 2
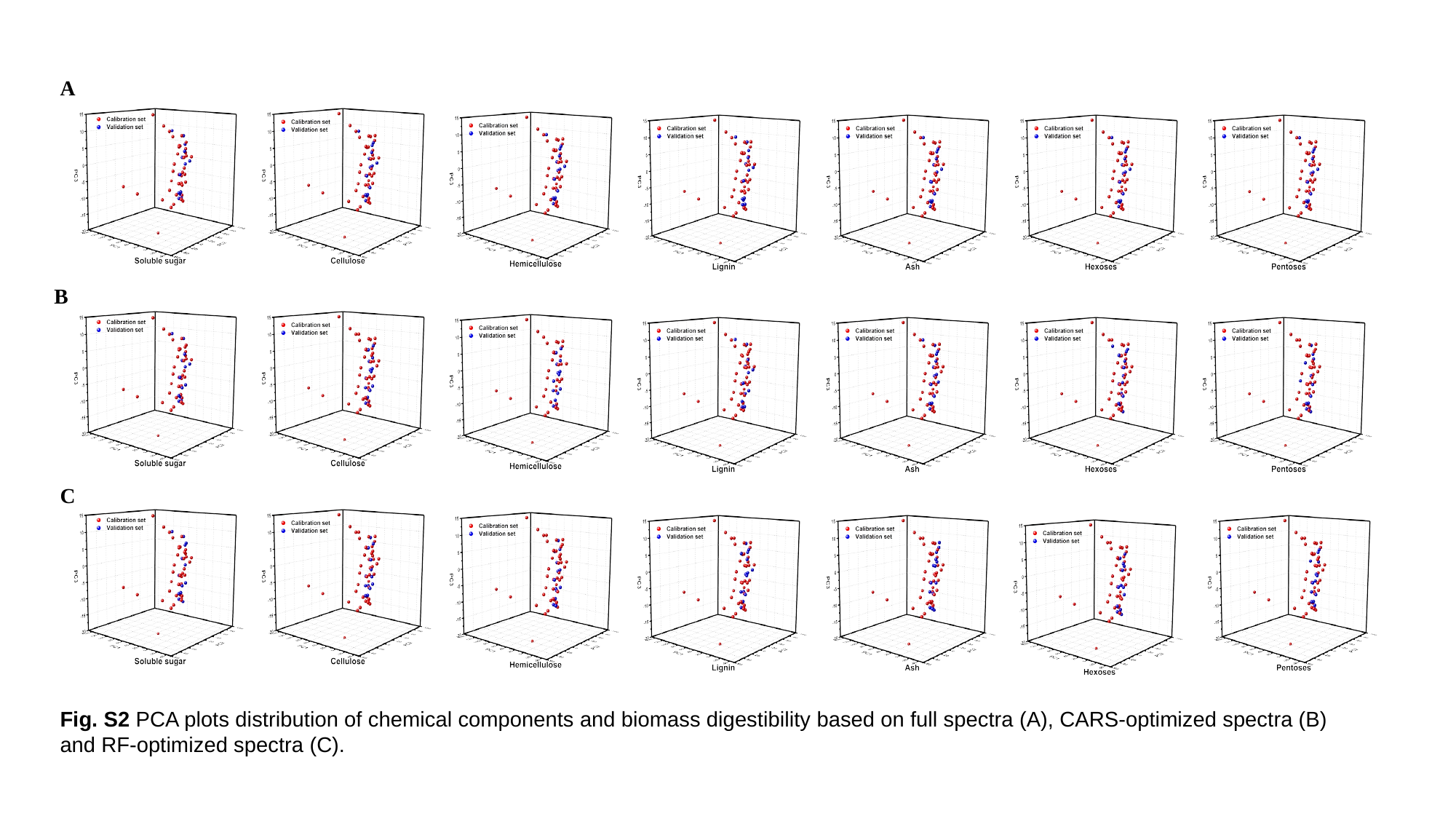

A
B
C
Fig. S2 PCA plots distribution of chemical components and biomass digestibility based on full spectra (A), CARS-optimized spectra (B) and RF-optimized spectra (C).

## Slide 3
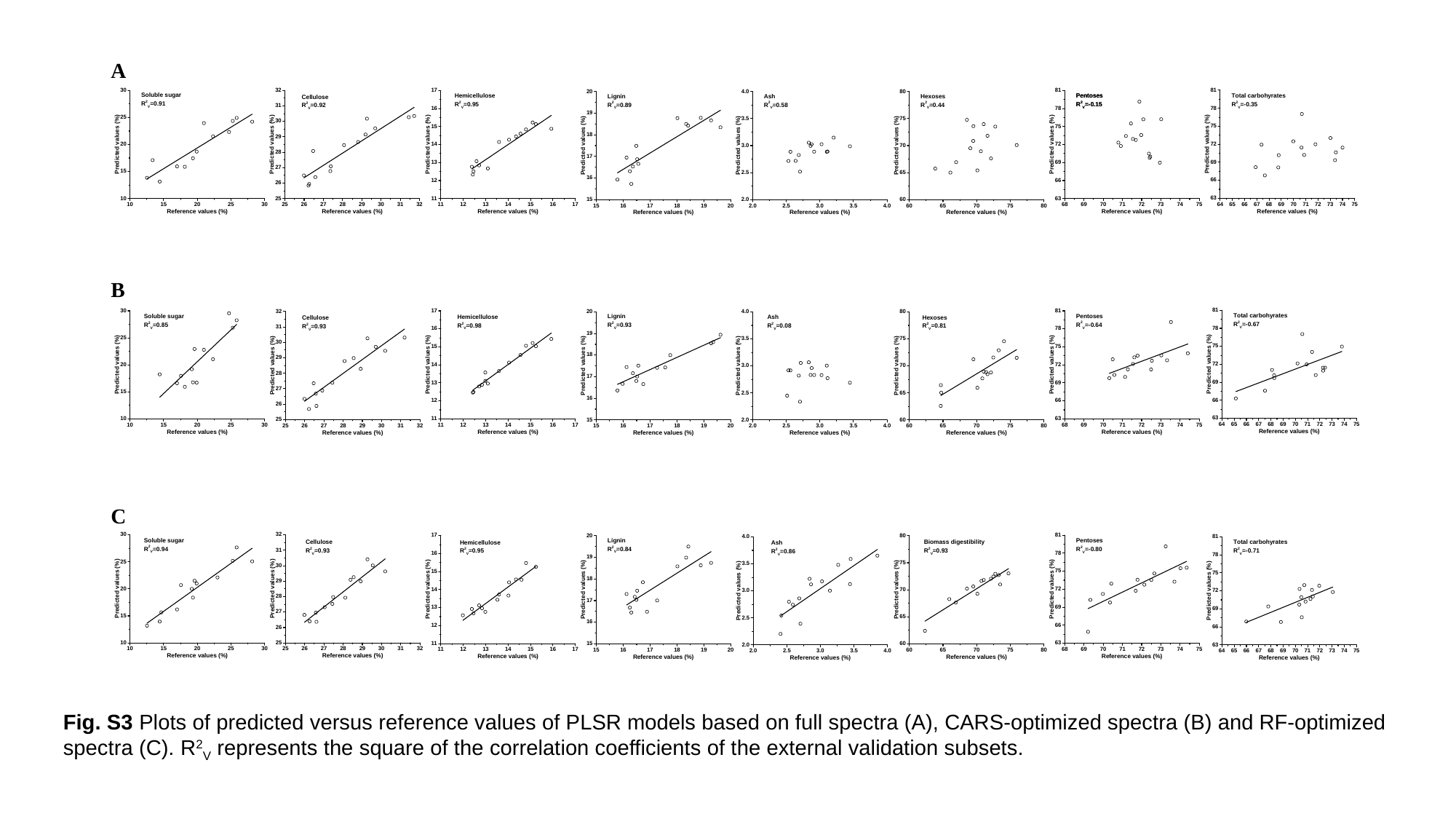

A
B
C
Fig. S3 Plots of predicted versus reference values of PLSR models based on full spectra (A), CARS-optimized spectra (B) and RF-optimized spectra (C). R2V represents the square of the correlation coefficients of the external validation subsets.

## Slide 4
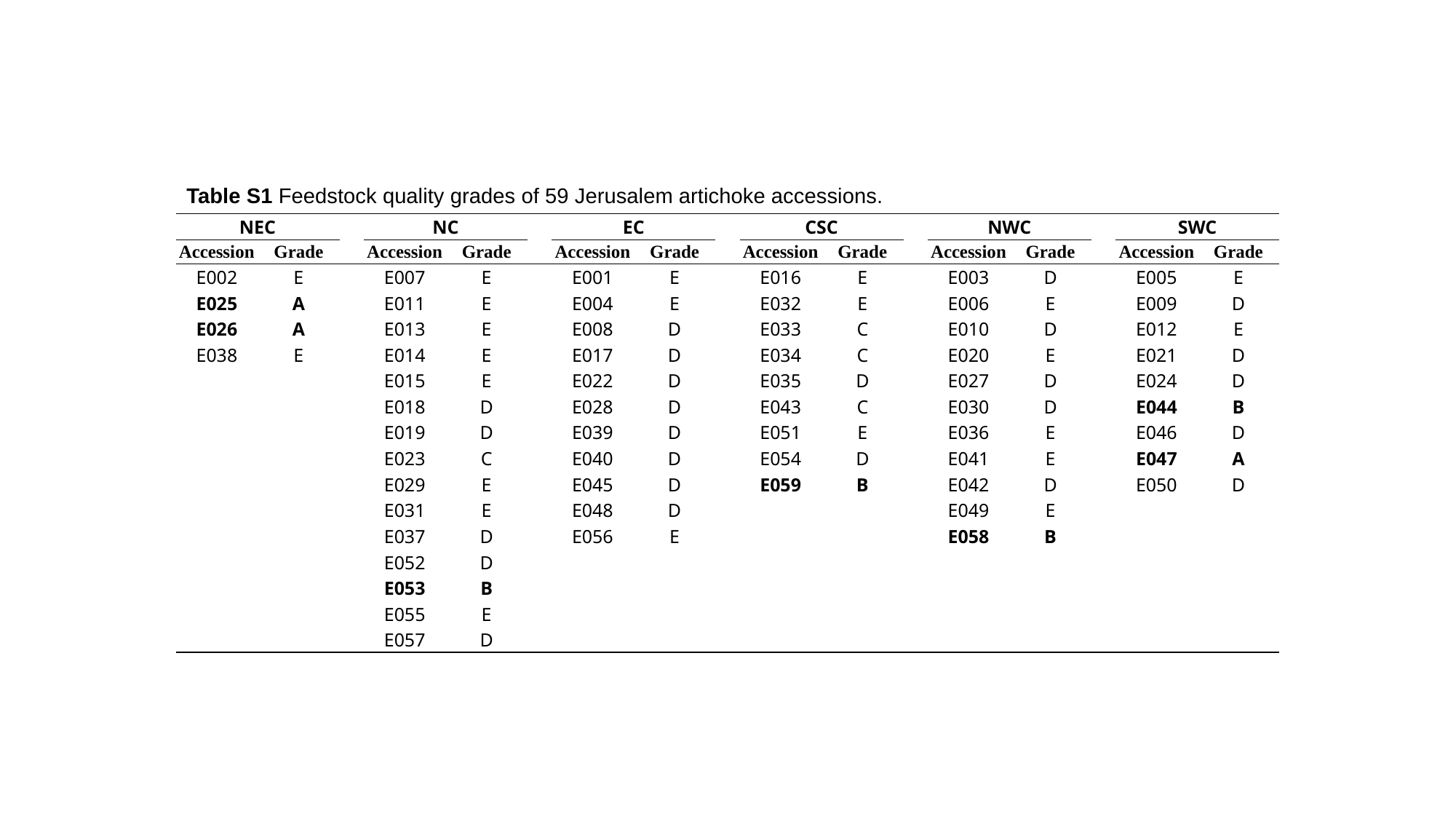

Table S1 Feedstock quality grades of 59 Jerusalem artichoke accessions.
| NEC | | | NC | | | EC | | | CSC | | | NWC | | | SWC | |
| --- | --- | --- | --- | --- | --- | --- | --- | --- | --- | --- | --- | --- | --- | --- | --- | --- |
| Accession | Grade | | Accession | Grade | | Accession | Grade | | Accession | Grade | | Accession | Grade | | Accession | Grade |
| E002 | E | | E007 | E | | E001 | E | | E016 | E | | E003 | D | | E005 | E |
| E025 | A | | E011 | E | | E004 | E | | E032 | E | | E006 | E | | E009 | D |
| E026 | A | | E013 | E | | E008 | D | | E033 | C | | E010 | D | | E012 | E |
| E038 | E | | E014 | E | | E017 | D | | E034 | C | | E020 | E | | E021 | D |
| | | | E015 | E | | E022 | D | | E035 | D | | E027 | D | | E024 | D |
| | | | E018 | D | | E028 | D | | E043 | C | | E030 | D | | E044 | B |
| | | | E019 | D | | E039 | D | | E051 | E | | E036 | E | | E046 | D |
| | | | E023 | C | | E040 | D | | E054 | D | | E041 | E | | E047 | A |
| | | | E029 | E | | E045 | D | | E059 | B | | E042 | D | | E050 | D |
| | | | E031 | E | | E048 | D | | | | | E049 | E | | | |
| | | | E037 | D | | E056 | E | | | | | E058 | B | | | |
| | | | E052 | D | | | | | | | | | | | | |
| | | | E053 | B | | | | | | | | | | | | |
| | | | E055 | E | | | | | | | | | | | | |
| | | | E057 | D | | | | | | | | | | | | |

## Slide 5
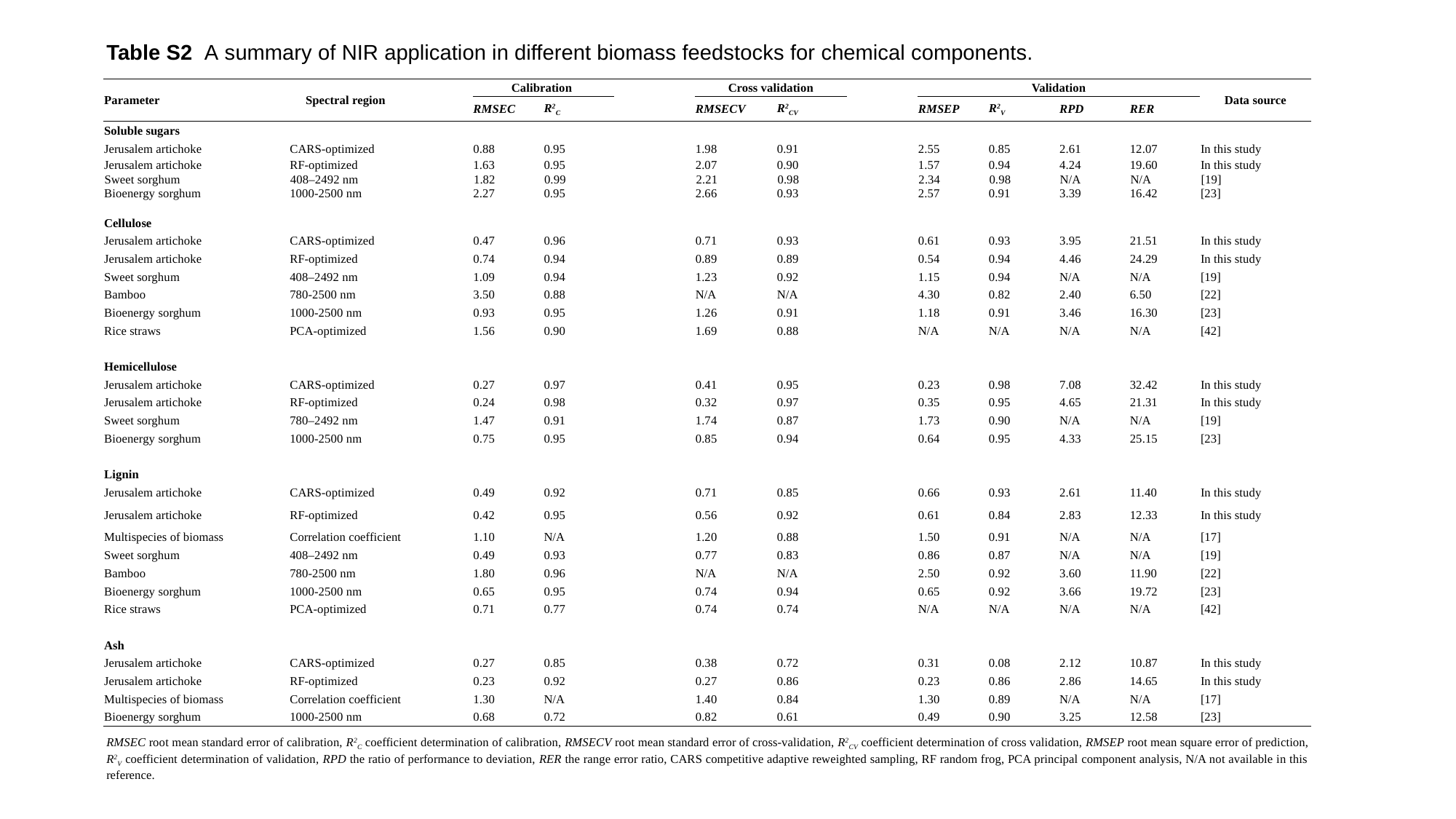

Table S2 A summary of NIR application in different biomass feedstocks for chemical components.
| Parameter | | Spectral region | | Calibration | | | Cross validation | | | Validation | | | | Data source |
| --- | --- | --- | --- | --- | --- | --- | --- | --- | --- | --- | --- | --- | --- | --- |
| | | | | RMSEC | R2C | | RMSECV | R2CV | | RMSEP | R2V | RPD | RER | |
| Soluble sugars | | | | | | | | | | | | | | |
| Jerusalem artichoke | | CARS-optimized | | 0.88 | 0.95 | | 1.98 | 0.91 | | 2.55 | 0.85 | 2.61 | 12.07 | In this study |
| Jerusalem artichoke | | RF-optimized | | 1.63 | 0.95 | | 2.07 | 0.90 | | 1.57 | 0.94 | 4.24 | 19.60 | In this study |
| Sweet sorghum | | 408–2492 nm | | 1.82 | 0.99 | | 2.21 | 0.98 | | 2.34 | 0.98 | N/A | N/A | [19] |
| Bioenergy sorghum | | 1000-2500 nm | | 2.27 | 0.95 | | 2.66 | 0.93 | | 2.57 | 0.91 | 3.39 | 16.42 | [23] |
| Cellulose | | | | | | | | | | | | | | |
| Jerusalem artichoke | | CARS-optimized | | 0.47 | 0.96 | | 0.71 | 0.93 | | 0.61 | 0.93 | 3.95 | 21.51 | In this study |
| Jerusalem artichoke | | RF-optimized | | 0.74 | 0.94 | | 0.89 | 0.89 | | 0.54 | 0.94 | 4.46 | 24.29 | In this study |
| Sweet sorghum | | 408–2492 nm | | 1.09 | 0.94 | | 1.23 | 0.92 | | 1.15 | 0.94 | N/A | N/A | [19] |
| Bamboo | | 780-2500 nm | | 3.50 | 0.88 | | N/A | N/A | | 4.30 | 0.82 | 2.40 | 6.50 | [22] |
| Bioenergy sorghum | | 1000-2500 nm | | 0.93 | 0.95 | | 1.26 | 0.91 | | 1.18 | 0.91 | 3.46 | 16.30 | [23] |
| Rice straws | | PCA-optimized | | 1.56 | 0.90 | | 1.69 | 0.88 | | N/A | N/A | N/A | N/A | [42] |
| | | | | | | | | | | | | | | |
| Hemicellulose | | | | | | | | | | | | | | |
| Jerusalem artichoke | | CARS-optimized | | 0.27 | 0.97 | | 0.41 | 0.95 | | 0.23 | 0.98 | 7.08 | 32.42 | In this study |
| Jerusalem artichoke | | RF-optimized | | 0.24 | 0.98 | | 0.32 | 0.97 | | 0.35 | 0.95 | 4.65 | 21.31 | In this study |
| Sweet sorghum | | 780–2492 nm | | 1.47 | 0.91 | | 1.74 | 0.87 | | 1.73 | 0.90 | N/A | N/A | [19] |
| Bioenergy sorghum | | 1000-2500 nm | | 0.75 | 0.95 | | 0.85 | 0.94 | | 0.64 | 0.95 | 4.33 | 25.15 | [23] |
| | | | | | | | | | | | | | | |
| Lignin | | | | | | | | | | | | | | |
| Jerusalem artichoke | | CARS-optimized | | 0.49 | 0.92 | | 0.71 | 0.85 | | 0.66 | 0.93 | 2.61 | 11.40 | In this study |
| Jerusalem artichoke | | RF-optimized | | 0.42 | 0.95 | | 0.56 | 0.92 | | 0.61 | 0.84 | 2.83 | 12.33 | In this study |
| Multispecies of biomass | | Correlation coefficient | | 1.10 | N/A | | 1.20 | 0.88 | | 1.50 | 0.91 | N/A | N/A | [17] |
| Sweet sorghum | | 408–2492 nm | | 0.49 | 0.93 | | 0.77 | 0.83 | | 0.86 | 0.87 | N/A | N/A | [19] |
| Bamboo | | 780-2500 nm | | 1.80 | 0.96 | | N/A | N/A | | 2.50 | 0.92 | 3.60 | 11.90 | [22] |
| Bioenergy sorghum | | 1000-2500 nm | | 0.65 | 0.95 | | 0.74 | 0.94 | | 0.65 | 0.92 | 3.66 | 19.72 | [23] |
| Rice straws | | PCA-optimized | | 0.71 | 0.77 | | 0.74 | 0.74 | | N/A | N/A | N/A | N/A | [42] |
| | | | | | | | | | | | | | | |
| Ash | | | | | | | | | | | | | | |
| Jerusalem artichoke | | CARS-optimized | | 0.27 | 0.85 | | 0.38 | 0.72 | | 0.31 | 0.08 | 2.12 | 10.87 | In this study |
| Jerusalem artichoke | | RF-optimized | | 0.23 | 0.92 | | 0.27 | 0.86 | | 0.23 | 0.86 | 2.86 | 14.65 | In this study |
| Multispecies of biomass | | Correlation coefficient | | 1.30 | N/A | | 1.40 | 0.84 | | 1.30 | 0.89 | N/A | N/A | [17] |
| Bioenergy sorghum | | 1000-2500 nm | | 0.68 | 0.72 | | 0.82 | 0.61 | | 0.49 | 0.90 | 3.25 | 12.58 | [23] |
RMSEC root mean standard error of calibration, R2C coefficient determination of calibration, RMSECV root mean standard error of cross-validation, R2CV coefficient determination of cross validation, RMSEP root mean square error of prediction, R2V coefficient determination of validation, RPD the ratio of performance to deviation, RER the range error ratio, CARS competitive adaptive reweighted sampling, RF random frog, PCA principal component analysis, N/A not available in this reference.

## Slide 6
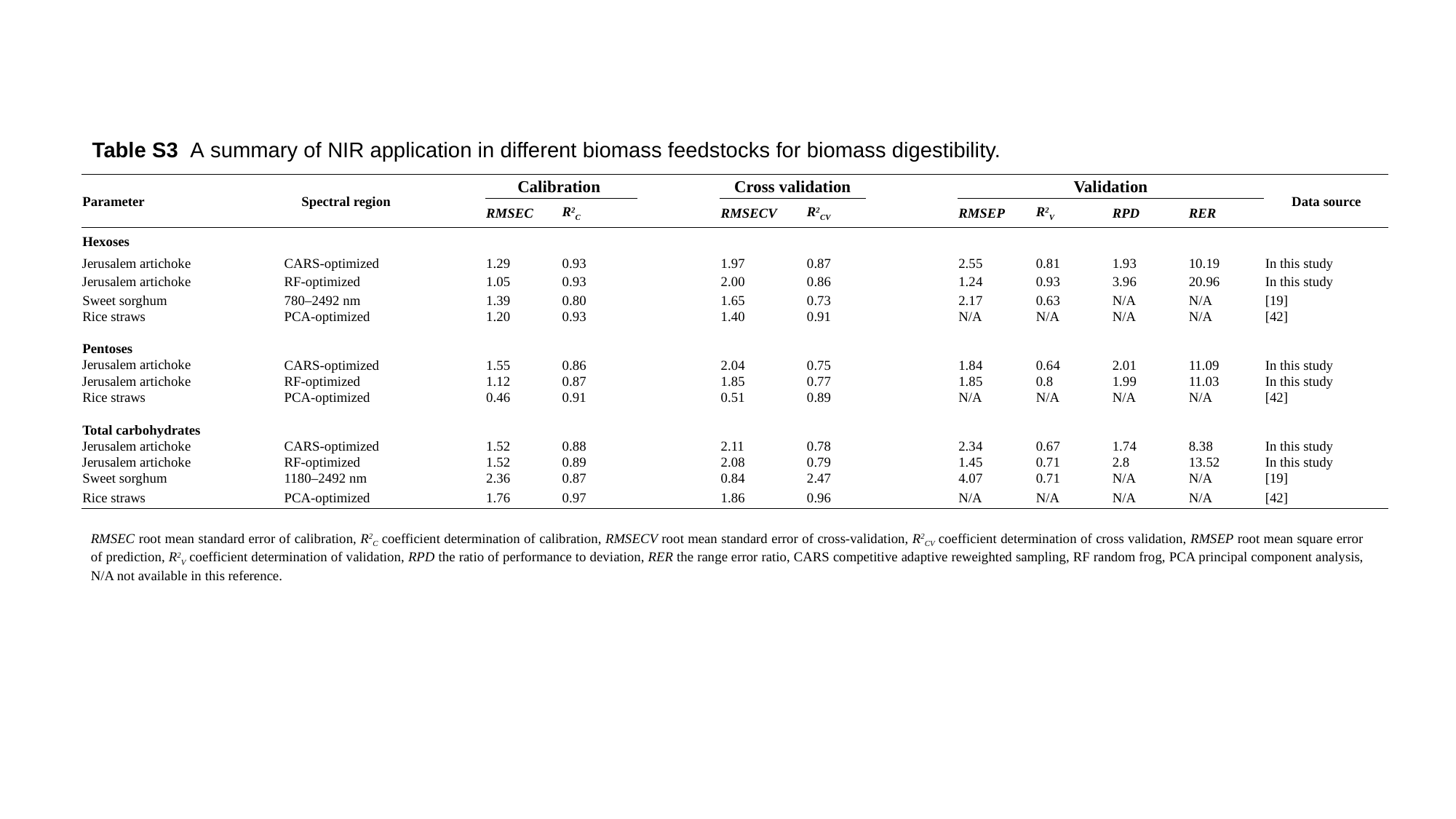

Table S3 A summary of NIR application in different biomass feedstocks for biomass digestibility.
| Parameter | | Spectral region | | Calibration | | | Cross validation | | | Validation | | | | Data source |
| --- | --- | --- | --- | --- | --- | --- | --- | --- | --- | --- | --- | --- | --- | --- |
| | | | | RMSEC | R2C | | RMSECV | R2CV | | RMSEP | R2V | RPD | RER | |
| Hexoses | | | | | | | | | | | | | | |
| Jerusalem artichoke | | CARS-optimized | | 1.29 | 0.93 | | 1.97 | 0.87 | | 2.55 | 0.81 | 1.93 | 10.19 | In this study |
| Jerusalem artichoke | | RF-optimized | | 1.05 | 0.93 | | 2.00 | 0.86 | | 1.24 | 0.93 | 3.96 | 20.96 | In this study |
| Sweet sorghum | | 780–2492 nm | | 1.39 | 0.80 | | 1.65 | 0.73 | | 2.17 | 0.63 | N/A | N/A | [19] |
| Rice straws | | PCA-optimized | | 1.20 | 0.93 | | 1.40 | 0.91 | | N/A | N/A | N/A | N/A | [42] |
| | | | | | | | | | | | | | | |
| Pentoses | | | | | | | | | | | | | | |
| Jerusalem artichoke | | CARS-optimized | | 1.55 | 0.86 | | 2.04 | 0.75 | | 1.84 | 0.64 | 2.01 | 11.09 | In this study |
| Jerusalem artichoke | | RF-optimized | | 1.12 | 0.87 | | 1.85 | 0.77 | | 1.85 | 0.8 | 1.99 | 11.03 | In this study |
| Rice straws | | PCA-optimized | | 0.46 | 0.91 | | 0.51 | 0.89 | | N/A | N/A | N/A | N/A | [42] |
| | | | | | | | | | | | | | | |
| Total carbohydrates | | | | | | | | | | | | | | |
| Jerusalem artichoke | | CARS-optimized | | 1.52 | 0.88 | | 2.11 | 0.78 | | 2.34 | 0.67 | 1.74 | 8.38 | In this study |
| Jerusalem artichoke | | RF-optimized | | 1.52 | 0.89 | | 2.08 | 0.79 | | 1.45 | 0.71 | 2.8 | 13.52 | In this study |
| Sweet sorghum | | 1180–2492 nm | | 2.36 | 0.87 | | 0.84 | 2.47 | | 4.07 | 0.71 | N/A | N/A | [19] |
| Rice straws | | PCA-optimized | | 1.76 | 0.97 | | 1.86 | 0.96 | | N/A | N/A | N/A | N/A | [42] |
RMSEC root mean standard error of calibration, R2C coefficient determination of calibration, RMSECV root mean standard error of cross-validation, R2CV coefficient determination of cross validation, RMSEP root mean square error of prediction, R2V coefficient determination of validation, RPD the ratio of performance to deviation, RER the range error ratio, CARS competitive adaptive reweighted sampling, RF random frog, PCA principal component analysis, N/A not available in this reference.
