# Supplementary material for: An NIRS-based assay of chemical composition and biomass digestibility for rapid selection of Jerusalem artichoke clones
Source: Biotechnol Biofuels. 2018 Dec 19;11:334. doi: 10.1186/s13068-018-1335-1 (PMC6299672; doi:10.1186/s13068-018-1335-1)
Supplement: Supplementary file 3 — Additional file 3. The procedure code of grey relational grade analysis. [file 13068_2018_1335_MOESM3_ESM.docx]

function [Score] = Score(x)

Mx = mean(x);

Ex = x./Mx;

Ox = max(Ex);

Ax = abs(Ex-Ox);

MAXAx = max(Ax(:));

MINAx = min(Ax(:));

P = 0.5

r = (MINAx+(P*MAXAx))./(Ax+(P*MAXAx))

Wc = input('chemical composition');

Wd = input(' biomass digestibility ');

[m,n] = size(x);

GRA = (mean(r(:,1:n-1),2)*Wc)+(r(:,n)*Wd);

Score = (GRA-min(GRA))./(max(GRA)-min(GRA))

end
